# A Rare Case of Complete Fragmentation of Pacemaker Lead after a High-Velocity Theme Park Ride

**DOI:** 10.1155/2018/4192964

**Published:** 2018-10-18

**Authors:** Furqan Khattak, Muhammad Khalid, Sathvika Gaddam, Vijay Ramu, Vipul Brahmbhatt

**Affiliations:** East Tennessee State University, Johnson City, TN, USA

## Abstract

Pacemaker lead fracture is one of the most common causes of pacemaker malfunction and is most frequently associated with weight lifting or chest trauma. These patients usually present with symptoms of dizziness, syncope, chest discomfort, and palpitations or less commonly with extracardiac symptoms. Diagnosis is made by ECG and careful review of chest imaging such as chest X-ray or fluoroscopy. Treatment involves placement of a new lead with or without extraction of the fractured lead. We present an interesting case of complete severance of the tip of a dual-chamber pacemaker's atrial lead after a high-velocity theme park ride. In our case, the fracture occurred during amusement park rides and went undiagnosed until the patient presented for routine pacemaker evaluation. This case indicates that extreme physical forces in the absence of direct trauma, such as during amusement park rides, may result in lead fractures and patients with pacemakers should be cautioned regarding such activities.

## 1. Introduction

Pacemaker lead fracture is one of the most common causes of pacemaker malfunction. Lead fractures are seen in approximately 0.1 to 4.2% of patients with pacemakers per year. Lead fractures are most commonly associated with physical exertion during weight lifting or chest trauma. Subclavian crush syndrome is one of the most commonly reported causes of traumatic pacemaker fractures. Stress or trauma due to physical exertion causes compression of a lead between the clavicle and first rib when implanted by subclavian approach. In our patient's case, fracture of a pacemaker lead occurred during accelerated rides in the absence of direct trauma.

## 2. Case Description

**Figure 1:**
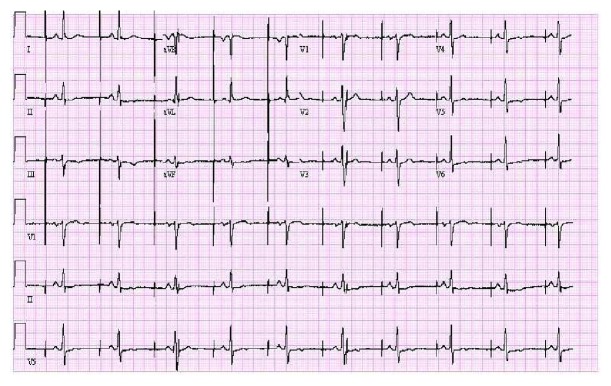
12-lead ECG showed normal sinus rhythm with loss of capture of atrial lead.

**Figure 2:**
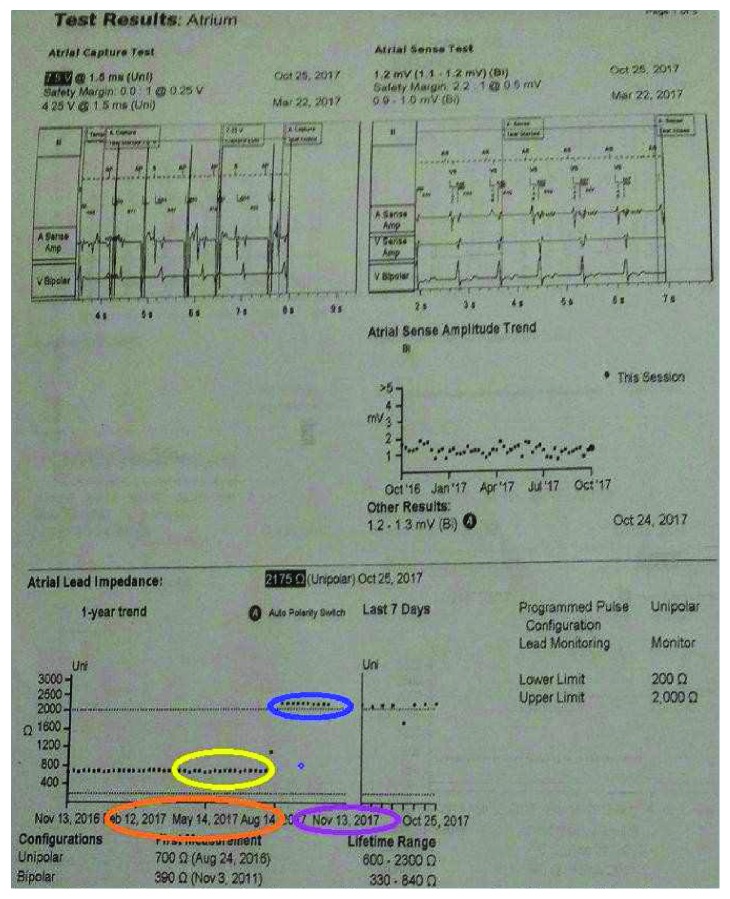
Device evaluation showed loss of atrial capture and sudden increase in lead impedance from normal in May 2017 (yellow circle) to 2000 ohms in August 2017 (blue circle).

**Figure 3:**
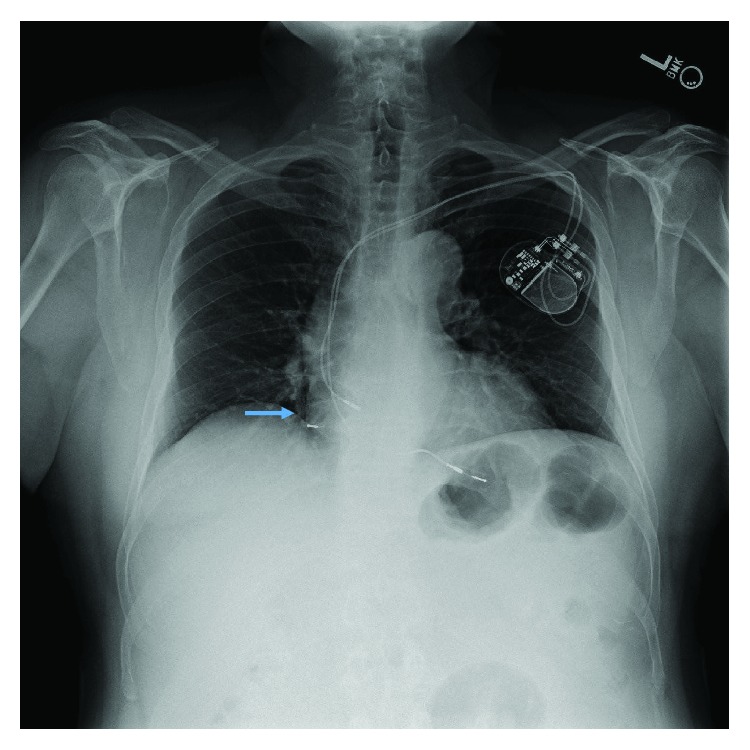
PA chest X-ray showed fractured right atrial lead (blue arrow).

**Figure 4:**
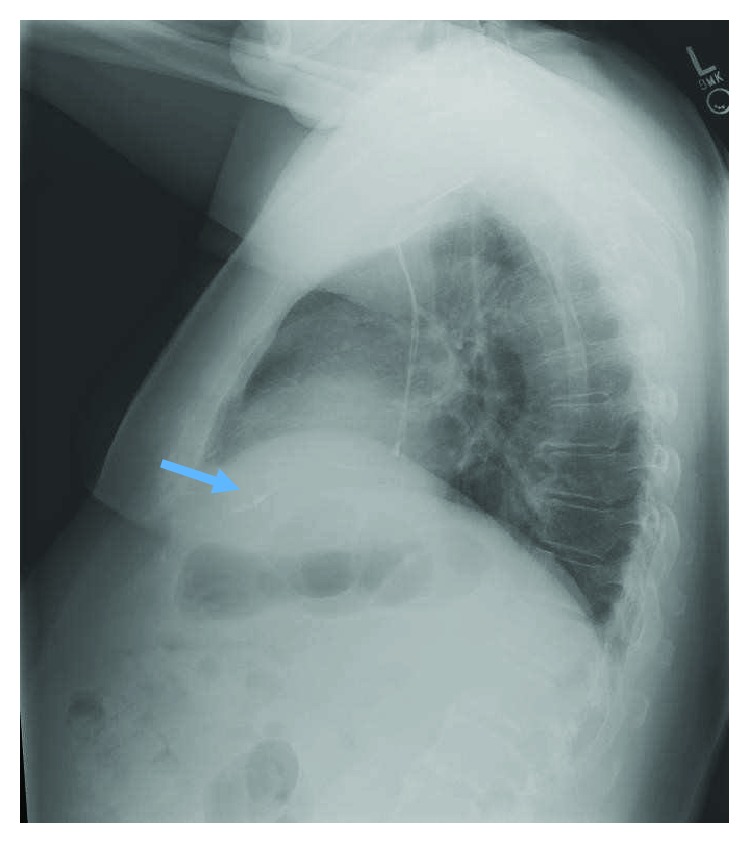
Lateral chest X-ray showed right atrial lead fracture (blue arrow).

A 62-year-old male with history of hypertension, coronary artery disease, and sick sinus syndrome presented to outpatient device check clinic to establish care for a pacemaker device. He underwent implantation of a dual-chamber pacemaker device (St. Jude Accent generator with Medtronic CapSure SP Novus atrial and ventricular leads) in 2002 for sick sinus syndrome and had a generator changeout in 2011. The lead model was not implicated in a recall to our knowledge and search. The patient had his last device check performed in March 2017, and no problems with the device function were reported at that time. The patient denied any trauma to the chest or upper extremities, chest pain, shortness of breath, palpitations, presyncope, or syncopal episodes. He denied any device-related complications in the past. The patient reported a recent visit to a popular theme park in the 1^st^ week of August, where he enjoyed multiple high thrill rides including high-velocity roller coaster rides. On physical examination, he was afebrile with normal pulse, blood pressure, and respiratory rate. His left pectoral pacemaker implant site showed no erythema, swelling, warmth, drainage, or signs of erosion. Labs showed normal blood counts and normal renal and liver function. A 12-lead ECG showed normal sinus rhythm with a heart rate of 60 beats per minute and atrial pacing spikes with loss of capture (Figure 1). Pacemaker device evaluation showed approximate remaining battery life of 9 years and programmed DDDR pacing mode. Heart rate histograms showed 54% atrial pacing and 15% ventricular pacing. The right ventricular lead showed normal sensing, impedance, and pacing threshold. The right atrial lead was noted to have unusually high impedance of 2175 ohms and no capture on testing at voltages as high as 7.5 mV. Lead impedance history clearly showed an abrupt increase in the atrial lead impedance in August, at the time patient had visited the theme park, from around 600 ohms to 1000 ohms and subsequently above 2000 ohms (Figure 2). EGM showed atrial lead noise. A chest X-ray (CXR) was obtained which revealed an intact right ventricular lead and complete severance of the right atrial lead tip (Figures 3 and 4). The pacemaker was reprogrammed from DDDR to VVIR mode. After discussion and review with the patient, it was decided not to retrieve the fractured lead or insert a new atrial lead, due to his intrinsic sinus rhythm having taken over, no evidence of atrioventricular (AV) block, and no current requirement for pacing. It was decided to follow the patient clinically to see if he will require insertion of an atrial lead in future.

## 3. Discussion

Pacemaker devices play an important role in the management of bradyarrhythmias, however, postimplantation complications can cause significant morbidity and mortality. Complications may occur during implant procedure, or, from pocket complications, generator malfunction, or lead failures. Pacemaker leads are considered the weakest link in this chain. Previous studies have reported lead-related complications such as lead dislodgement or lead damage during implantation, electrical failure, and lead fracture due to chest trauma or insulation defects in different pacemaker devices. Studies have also shown the benefit of postimplantation chest X-ray to look for immediate implantation-related complications, perforation, and malposition of leads. Early lead perforation is a rare complication of device implantation in the right atrium or right ventricle (0.1–0.8%) and occurs within one month of implantation. Delayed lead perforation occurs after the first month and is usually differentiated from early perforation by the absence of cardiac tamponade related to lead perforation. Lead fractures are seen with an incidence rate of 0.1 to 4.2% per patient year [1]. The most common site of lead fracture is at the site of entry (40%) followed by the area between the entry site and generator (28%) and lastly close to the generator site (23%). Only 7% are intravascular fractures [1]. Tricuspid valve has been reported as an unusual site of pacemaker lead fracture [2].

Most lead fractures occur in the pacemaker pocket or between clavicle and the first rib mainly due to compression of a lead lateral to the entry site between soft tissues muscles and ligaments. Pacemaker lead fracture due to physical exertion is an uncommon albeit recognized cause of lead malfunction. [3]. Few cases of traumatic lead fracture due to blunt chest trauma [4] and weight lifting due to crush injuries between clavicle and the first rib have been reported [5]. Subclavian crush syndrome is one of the most commonly reported causes of traumatic pacemaker fractures.

Studies have shown that fracture rate of multifilar coil leads is less than the single filar coil leads [6]. Lead fracture depends on factors such as coil diameter, radius of curvature, and approach used for implantation, with more fractures occurring with internal jugular vein approach than external jugular or cephalic vein approach. Usually, lead fracture is associated with high impedance, but there have been cases reported with normal impedance in multifilar coil lead fracture resulting from subclavian crush syndrome. The mechanism behind low impedance was a connection between inner and outer coil [7]. A case of lead fracture due to cardiac fibroma was also reported with low impedance [8]. Another case has been reported about loss of atrial pacing with possible atrial mode switch due to atrial oversensing in a patient with atrial arrhythmias [9].

Patients with a lead fracture may present with symptoms of dizziness, syncope, chest discomfort, and palpitations or less commonly extracardiac symptoms like hiccups or maybe completely asymptomatic as in our patient. Diagnosis is often made by ECG and careful review of chest imaging such as chest X-ray or fluoroscopy. Treatment involves placement of a new lead with or without extraction of the fractured lead. The decision regarding extraction of the old lead is individualized considering the age of the lead and expected fibrosis. Often, the old lead is capped and left alone to avoid complications of lead extraction such as arrhythmias and cardiac perforation.

Leadless pacemakers are now becoming available in the US market, and Micra, the first leadless pacemaker device, has been approved by FDA. Leadless devices implanted by transcatheter approach may theoretically reduce complications related to device implantation and those related to lead itself. This may be the future of pacing in suitable candidates who require only single chamber (right ventricle) pacing [10].

Our case is unusual to begin with as our patient had a dual-chamber pacemaker implanted for sick sinus syndrome at the age of 46 years; however, he did not show any progression to AV block after 16 years which is uncommon for molecular abnormalities of ion channel/cardiac proteins which usually undergo significant evolution to bradycardia/AV block with time. Second, to our knowledge, this is the first case of a pacemaker lead fracture associated with a theme park ride and indicates that widely popular high-velocity theme park rides may pose risks to pacemaker leads. We hypothesize that the sheer stress generated by extreme acceleration and gravitational forces on a wire that is moving with cardiac cycle during the high-velocity rides may cause fracture even in the absence of direct physical trauma. The site of fracture in this case, which is a transition from ring to tip electrode of the atrial lead, rules out any possible role of surrounding skeletal structures, such as in cases of rib/clavicle/pocket fractures. The lack of previous chest X-ray in our center precluded the comparison between the current lead position and the position at implantation, which in fact could influence the fracture. Therefore, it cannot be inferred whether the lead tip was at the anterior-lateral location as the X-ray shows or if it migrated to this location after the lead body was snapped in two parts. This case may have important clinical implications such as the need for caution with theme park rides, and routine pacemaker interrogations after such visits especially in pacemaker-dependent patients. Luckily, our patient remained unharmed as he had intact intrinsic AV nodal conduction and required infrequent atrial pacing. Moreover, at the time of presentation, he had intrinsic sinus activity and intact AV nodal conduction and the failed atrial pacing programmed at 60 bpm was redundant.
